# Spatial and temporal groundwater biogeochemical variability help inform subsurface connectivity within a high-altitude Alpine catchment (Riale di Ronco, Switzerland)

**DOI:** 10.3389/fmicb.2025.1522714

**Published:** 2025-02-25

**Authors:** Andrew S. Acciardo, Moira Arnet, Nima Gholizadeh Doonechaly, Alberto Ceccato, Paula Rodriguez, Hoang N.H. Tran, Quinn Wenning, Eric Zimmerman, Marian Hertrich, Bernard Brixel, Cara Magnabosco

**Affiliations:** ^1^ETH Department of Earth and Planetary Sciences, Zurich, Switzerland; ^2^Centre for Hydrogeology and Geothermics (CHYN), Neuchâtel, Switzerland; ^3^Deloitte, Zurich, Switzerland; ^4^Cantonal Office of the Environment, Geneva, Switzerland

**Keywords:** subsurface microbiology, underground laboratory, groundwater, microbial ecology, deep life observatory

## Abstract

Accessing the deep terrestrial subsurface (greater than 1 km below the surface) presents significant practical challenges, leaving these ecosystems largely uncharacterized despite their extensive presence beneath Earth's landmasses. In this study, we introduce the BedrettoLab Deep Life Observatory (DELOS), a new underground laboratory to study the biogeochemical diversity of groundwater in a high-altitude Alpine catchment tens of meters to 1.6 km underground. Biogeochemical monitoring of DELOS over spatial and temporal scales highlight three dominant ecotypes throughout DELOS: (1) Shallow groundwater with low electrical conductivity enriched in *Leptospirillia*; (2) High-inflow fault zones enriched in ultra-small bacteria and archaea; (3) Bicarbonate-enriched waters that are enriched in *Candidatus* Kryptonia and *Spirochaetota*. Despite a consistent lithology throughout DELOS, groundwater from fractures that are spatially near each other are not always represented by the same ecotype and can be more similar to groundwater emitted from fractures thousands of meters away. Despite this heterogeneity, the biological and hydrochemical compositions of the groundwater of individual fractures remained relatively stable throughout the course of a 1-year monitoring period. An exception to this trend occurred after a series of seismic events near one groundwater-bearing fracture. Here, the microbial community and hydrochemical composition of the groundwater changed after the seismic events but returned to the site's “baseline” composition within 3 weeks. Taken together, these findings provide new insights into the spatial and temporal heterogeneity of deep subsurface ecosystems and the subsurface connectivity of an Alpine subsurface environment.

## 1 Introduction

Since the earliest reports of subsurface life in coal beds and oil fields in the 1920s (Bastin et al., 1926; Ginsburg-Karagitscheva, 1926; Lipman, 1928), the field of subsurface geomicrobiology has grown significantly (for a review, see Onstott, 2016) and it is now estimated that more bacteria and archaea reside in the continental subsurface than any other microbial habitat (for a review, see Magnabosco et al., 2018a). All three domains of life and even animals have been found to be capable of surviving hundreds of meters to kilometers underground (Borgonie et al., 2015) and, in many cases, survive exclusively through the utilization of geologically-generated energy sources such as H_2_ and CO2 (for a review, see Magnabosco et al., 2019). Due to the practical challenges of accessing and studying deep (>500 m) subsurface ecosystems, a variety of subsurface experiments and monitoring programs have been pursued in underground laboratories such as the Äspö Hard Rock Laboratory (Äspö HRL, Sweden) (e.g. Kotelnikova and Pedersen, 1998; Pedersen, 1997; Kyle et al., 2008; Westmeijer et al., 2022), Mizunami Underground Research Laboratory (Japan) (e.g. Fukuda et al., 2010; Ino et al., 2016), Grimsel Test Site (Switzerland) (e.g. Konno et al., 2013), Mont Terri Underground Rock Laboratory (Switzerland) (e.g. Stroes-Gascoyne et al., 2007; Poulain et al., 2008; Bagnoud et al., 2016) and Sanford Underground Research Facility (USA) (e.g. Osburn et al., 2019; Casar et al., 2020; Momper et al., 2023). These research programs have revealed diverse communities of subsurface microorganisms capable of performing a wide range of biogeochemical functions such as methane, nitrogen, sulfur, and iron cycling (e.g. Pedersen, 1997; Fukuda et al., 2010; Konno et al., 2013; Ino et al., 2016; Wu et al., 2017; Lopez-Fernandez et al., 2018, 2023). This study describes the BedrettoLab Deep Life Observatory (DELOS), a new granite-hosted underground laboratory for subsurface microbiology investigations operated by ETH Zürich and located within the Bedretto Tunnel (Switzerland).

The Bedretto Tunnel was originally constructed as an adit for Furka Base Railway Tunnel from 1971 to 1982 and intersects 3 geological units in the Gotthard massif (e.g. Hafner, 1958; Keller and Schneider, 1982; Rast et al., 2022): (1) the Tremola Series from tunnel meter (TM) 0 to TM-434; (2) the Prato Series (TM-434 to TM-1138); and (3) the Rotondo granite (TM-1138 to TM-5218). The metasedimentary Tremola Series is composed of mica-gneiss, amphibolites, schists, calc-silicate rocks, and quartzites (Hafner, 1958; Keller and Schneider, 1982) that change between rock types over meter scales (Labhart, 2005). The Prato Series also exhibits compositional heterogeneity of mica-gneisses, amphibolites, and schists over centimeter to meter scales (Labhart, 2005). A sharp contact between the Prato Series and Rotondo granite occurs at TM-1138 and large amounts of deformation in the granite is observed between TM-1,138 and TM-1,143 (Rast et al., 2022). The Rotondo granite is dissected by brittle faults (Lützenkirchen, 2002) and ductile shear zones (Rast et al., 2022) and is further divided into a biotite-rich porphyritic granite (RG2) in the region of TM-2,800 to TM-3,440 that is surrounded by an equigranular granite (RG1) composed of quartz, alkali feldspar, plagioclase, and biotite (Rast et al., 2022). ^206^Pb/^238^U Zircon dating of the Rotondo granite indicates intrusion ages of 285 to 319 Ma for RG1 and 280 to 335 Ma RG2. Groundwater residence time estimates based on tritium for natural fractures spanning approximately TM-3,800 to TM-5,218 indicate that glacially-fed fractures experience rapid recharge (1 to 1.5 years) while non-glacially-fed fractures exhibit a much more complex recharge history and contain a sub-modern component (Ofterdinger, 2001). It is worth noting that these hydrochemical measurements are limited to the mineralogically consistent last ~1,500 m of the Bedretto Tunnel due to a tunnel collapse that occurred while the tunnel was left un-maintained from 1982 to 2015 and the residence time of groundwater from TM 0 − 3, 800 is currently unknown.

In 2018, ETH Zürich opened the “Bedretto Underground Laboratory for Geosciences and Geoenergies” (BULGG) between the Bedretto Tunnel's TM-2,000 and TM-2,100 (Ma et al., 2022). The first deep (197–303 m in length) boreholes of the BedrettoLab were drilled in 2019 and ahead of the “Bedretto Reservoir Project” (BRP; May 2021 to July 2023) which executed a series of hydraulic stimulation experiments designed to aid in the development of engineered geothermal systems (Gischig et al., 2020; Ma et al., 2022). In preparation for the BRP hydraulic stimulations, the BedrettoLab Deep Life Observatory (DELOS) was initiated to study the subsurface microorganisms residing within the Gotthard Massif. DELOS was named as an homage to the ancient Athenian island that prohibited death, due to the slow turnover times observed in the continental subsurface (Onstott et al., 2014). Since the majority of the Bedretto Tunnel is not cemented, DELOS is not restricted to the experimental boreholes of BULGG. Instead, inflows from the densely fractured Rotondo granite and gneissic sections of the 5.2 km Bedretto Tunnel can be easily sampled and provide an in-depth view of fractured rock ecosystems from tens to ~1,600 meters below land surface (mbls). This study describes the microbiological and hydrochemical changes throughout the natural fractures of the Bedretto Tunnel over a 1-year period from 2020 to 2021 and captures a period ahead of, during, and after the first hydraulic stimulation experiments within BULGG.

## 2 Materials and methods

### 2.1 Area of Study

**Figure 1:**
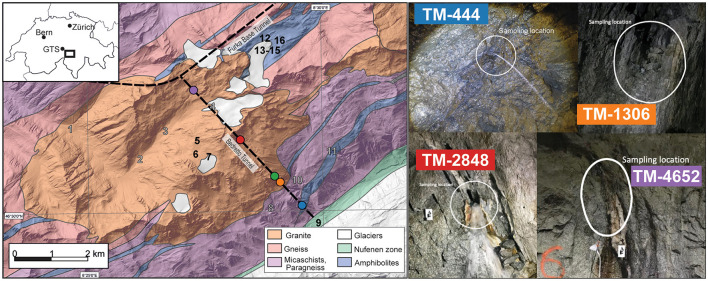
A geologic map (modified after Rast et al., 2022) of the BedrettoLab Deep Life Observatory (DELOS) is shown on the left of the figure and overlayed with the location of the Bedretto Tunnel and Furka Base Tunnel (dotted black lines). Tunnel Meter (TM) 0 occurs at the Val Bedretto tunnel entrance. The locations of time series sampling sites TM-444 (blue), TM-1306 (orange), TM-1494 (green), TM-2848 (red), and TM-4562 (purple) within the Bedretto Tunnel are illustrated as circles. Numbers 1–16 on the geologic map indicate surface water sampling sites. Gray numbers indicate locations where hydrochemistry samples were collected and black numbers indicate locations where DNA and hydrochemistry samples were collected. Notable surface DNA sample locations are the Ticino River (9), a high-Alpine spring (7), glacial lakes (5, 6, 12, 16), and the Witenwasseren glacier borehole (13) and flow (14, 15). A map of Switzerland (top left) indicates the locations of DELOS, the Grimsel Test Site (GTS), Zürich, and Bern. Four representative images of groundwater sampling sites are shown on the right. The white circles indicate the groundwater inflow that was sampled for this study. A complete image catalog of sampling sites can be found in Supplementary Data 1.

Surface water from above the Bedretto Tunnel (Figure 1) and groundwater from the BedrettoLab Deep Life Observatory (DELOS) and Grimsel Test site (GTS, https://grimsel.com/) were collected for this study. DELOS is accessed through the Ronco Portal of the Bedretto Tunnel (46.497518°N, 8.494992°E) in the Gotthard Massif (Switzerland, Figure 1) and is part of the Riale di Ronco Alpine catchment. The far end of the Bedretto Tunnel (Furka Portal) intersects the 15 km long Furka Base Tunnel. Only a few sections across the tunnel have been shotcreted or supported by steel arches (structural support is mainly required near the Ronco Portal), providing a unique opportunity to sample groundwater directly from fractures spanning over 5 km (Figure 1, Supplementary Data 1). The GTS is an underground research laboratory located approximately 15 km northwest of DELOS and situated within the granitic rock of the Aarmassif (Keusen et al., 1989). The GTS has been in operation since 1984 and has supported a variety of field-scale experiments related to radioactive waste disposal and enhanced geothermal systems. The similar geology and proximity of GTS to DELOS provided motivation to collect, analyze, and compare the microbial communities and water composition of GTS boreholes “Pinkel”, “ISC Injection 2” and “SB80001”.

### 2.2 Field Measurements And Sampling Strategy

DELOS groundwater samples were obtained from a total of 57 locations. 54 sampling sites were related to natural water inflows along the Bedretto tunnel and 3 sites were connected to tunnel inflows in smaller, uncased boreholes (Ø 10 cm, length unknown) in the tunnel sidewalls (Tunnel Meter (TM)-901, TM-755A and TM-2794, where TM refers to the distance in meters from the Val Bedretto tunnel entrance). Approximately monthly hydrochemical sampling from August 2020 to December 2021 and biological sampling from November 2020 to December 2021 are reported in this study. Additional samples were collected from three boreholes in the Grimsel Test Site in April 2021 and a variety of surface water sources in the DELOS region during the summer of 2021 (Figure 1). Temperature, pH, and electrical conductivity (EC) were directly measured on site using an electronic field probe (EXTECH, multi-parameter PCSTestr 35) that was 3-point calibrated (pH: 4.01, 7.00 and 10.00; EC: 84 μS cm−1, 147 μS cm−1 and 1413 μS cm−1). The redox potential (ORP) was measured with a KBM-100 (Voltcraft) field meter. The total alkalinity (carbonate alkalinity) of samples collected from November 2020 to March 2021 was determined through acid titration (Hach^®^, AL-AP Test) and flow rates (Q) were measured using a bucket-and-stopwatch approach.

### 2.3 Hydrochemical analyses

For major ion analysis, 2 × 5 mL filtered water samples were collected in the field, transported on ice and stored at 4°C before cation (Mg^2+^, Ca^2+^, K^+^, Na^+^, ${\text{NH}}_{4}^{+}$, Li^+^, Sr^2+^) and anion (${\text{SO}}_{4}^{2-}$, Cl^−^,F^−^, ${\text{NO}}_{3}^{-}$, ${\text{NO}}_{2}^{-}$, ${\text{PO}}_{4}^{3-}$, Br^−^) analysis via ion chromatography (IC; Dionex DX-120 IC System) at the Institute of Geological Sciences ETH Zürich. Prior to ion analysis, the EC and pH of each sample was measured using the inoLab EC-pH measuring instrument from WTW. Samples that exceeded the IC working range of 0-500 μS cm^−1^ were diluted 10× to ensure ion concentrations were within the ranges of the standards. Water isotope samples were collected and transported to Zürich in 100 mL polyethylene bottles with airtight caps (Semadeni, CH). In Zürich, water isotope samples were filtered using a 0.45 μm filter, sealed in 3 × 2 mL glass autosampler vials and stored at 4°C until water isotope analysis was performed using a Picarro L2130-i analyzer at the Geological Institute of ETH Zürich. Raw measurements were post-processed using the Picarro software ChemCorrect which calculates the isotopic composition as δ^18^O or δ^2^H-value with respect to the Vienna Standard Mean Ocean Water (VSMOW). The local meteoric water line of DELOS was estimated from regional isotope data obtained on waterisotopes.org and derived from (Bowen and Revenaugh, 2003).

### 2.4 DNA extraction, 16S rRNA gene amplification, quantification, and sequencing

Biomass was collected on site by filtering fracture or surface water through 0.22 μm Sterivex filters (SVGPL10RC, Millipore) with a peristaltic pump. Prior to filtration, new sterile tubing was flushed with the fracture water of interest for 3–5 minutes. After filtration, Sterivex filters were stored in sterile, 50 mL falcon tubes, transported on ice to ETH Zürich the same day, and frozen at -80°C until DNA extraction. DNA extractions of samples and extraction blanks were performed following the manufacturer's standard protocol of the DNeasy PowerWater Kit (14900-100-NF, Qiagen). DNA concentrations were quantified by the Qubit 4 Fluorometer (ThermoFisher) and stored in a -80°C freezer for use in downstream applications.

All DNA samples were amplified in triplicate using the Platinum II Hot-Start PCR Master Mix Kit (14000013, Invitrogen) and following a previously defined procedure (McNichol et al., 2021). Briefly, a single-step PCR was performed to amplify a conserved part of the V4-V5 region of the 16S SSU rRNA gene from bacteria and archaea. The region was targeted using Golay-barcoded 515F forward primers (AATGATACGGCGACCACCGAGATCTACACGCT - XXXXXXXXXXXX - TATGGTAATTGT - GTGYCAGCMGCCGCGGTAA, with the X's denoting the variable Golay barcode location) and a 926R PCR reverse primer (CAAGCAGAAGACGGCATACGAGATAGTCAGCCAGGG - CCGYCAATTYMTTTRAGTTT) (Walters et al., 2016). After PCR, triplicates were pooled together and samples were cleaned using Agencourt AMPure XP beads (Beckman Coulter) at a 0.7× ratio and DNA concentrations were measured using the Qubit 4 Fluorometer (ThermoFisher). Using the Qubit measured amplicon concentrations, samples were normalized and pooled. The pooled library was cleaned with AMPure XP beads at a 0.7× ratio and the final, cleaned library was quantified using both the Qubit 4 and 4150 Tapestation (Agilent). The amplicon library was sequenced at the ETH Zürich Genetic Diversity Center on an Illumina MiSeq using a 500-cycle paired end V2 reagent kit (MS-102-2003, Illumina). PhiX was spiked in at a concentration of 8%. The final library loading concentration was 4 pM and custom sequencing primers were used: 515Fseq (TATGGTAATTGTGTGYCAGCMGCCGCGGTAA), 926Rseq (CGGCATACGAGATAGTCAGCCAGGGCCGYCAATTYMTTT RAGTTT), and a modified Illumina index sequence primer (AATGATACGGCGACCACCGAGATCTACACGCT).

16S rRNA gene amplicon copy numbers were quantified using a quantitative PCR (qPCR) with the Lightcycler 480 (Roche). Briefly, a solution containing the V4-V5 forward primer 515F (GTGNCAGCMGCCGCGGTAA, final concentration 0.5 μM), the V4-V5 reverse primer 926R (CCGYCAATTYMTTTRAGTTT, 0.5 μM), 5 μL SsoFast EvaGreen Supermix with Low ROX 2x (1725210, BioRad), and 1 μL Bovine Serum Albumin (10 mg mL^−1^) were combined with 2 μL of template DNA were prepared in triplicate and loaded onto a 96-well plate for qPCR. 16S rRNA gene standards ranging from 2.088 × 10^6^ to 20.88 16S rRNA gene amplicon copies per μL were prepared using a consensus 16S synthetic construct and serial dilution. The qPCR program included an initialization of 95°C for 300 seconds, 40 cycles of denaturation (10 seconds at 95°C), annealing (30 seconds at 50°C) and elongation (15 seconds at 68°C) with a melting curve of 15 seconds at 95°C and 60 seconds at 55°C and one acquisition per °C from 55°C to 95°C. The fit (*R*^2^ = 0.99) and melt curve of the standards are shown in Supplementary Figures 1, 2.

### 2.5 Biodiversity analyses

The quality of 16S rRNA gene amplicon sequences was assessed using FastQC (Babraham Bioinformatics Group). Based on the sequence quality, the first 5 nucleotides of the forward and reverse reads were trimmed using the FASTX-trimmer, http://hannonlab.cshl.edu/fastx_toolkit/license.html. Additionally, nucleotides beyond position 230 were removed from the reverse read by the FASTX-trimmer. After trimming, Trimmomatic (Bolger et al., 2014) was used to quality filter the reads (SLIDINGWINDOW:100:28) and remove adapters. Paired end reads were joined using fastq-join (-p 3 -m 20; v-1.04.807), https://github.com/ExpressionAnalysis/ea-utils/blob/wiki/FastqJoin.md. Amplicon sequencing variants (ASVs) were then predicted from joined reads using the “pseudopool“ option of dada2 (Callahan et al., 2016) and annotated using dada2 and the Silva nr99 v138.2 dataset (Quast et al., 2012; Yilmaz et al., 2014). After annotation, all ASVs found in the control samples were removed from the ASV data table and omitted from all downstream analyses. Biodiversity and statistical analyses were performed in R and python with ASV data. All code is available via https://github.com/GeobiologyLab/DELOS-2021-time-series.

## 3 Results

### 3.1 Seasonal and spatial hydrochemical variability throughout DELOS

**Figure 2:**
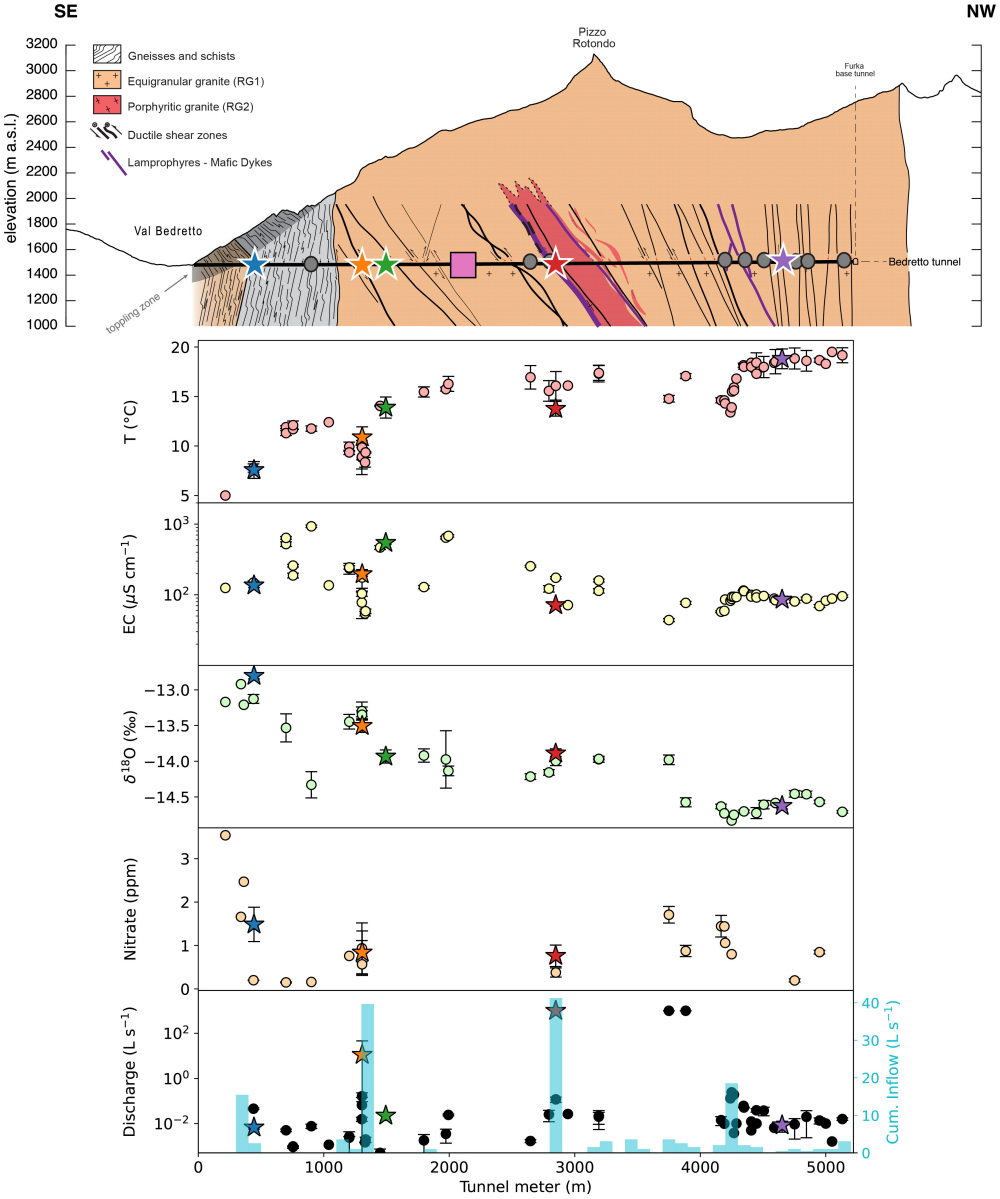
**Top:** A profile of the BedrettoLab Deep Life Observatory (DELOS, horizontal black line) is illustrated with respect to its overburden (modified after Rast et al., 2022). Notable geological features throughout DELOS are described in the panel legend and the Bedretto Underground Laboratory for Geosciences and Geoenergies is indicated by a pink box. Gray circles and colored stars along the tunnel indicate locations where DNA samples were collected as either single samples (gray circles) or as part of the “long-term biological monitoring” program (colored stars). **Lower:** The mean (points) and standard deviation (bars) of temperature (T), electrical conductivity (EC), δ^18^O, nitrate (samples whose concentrations were below detection are omitted), and discharge (black) measurements of individual fractures throughout a 1-year time series are displayed. Fractures with flow rates above what could be quantified with our measurement protocol were designated as 999 L s^−1^ (TM-2848, TM-3750, TM-3884). To better illustrate tunnel inflow from groundwater-bearing fractures, the cumulative discharge rate binned per 100 m interval is shown (cyan bars, previously reported by Masset and Loew (2010)). m.a.s.l. = meters above sea level.

DELOS groundwaters exhibit annual mean temperatures from 5°C to 19.5°C and a pH ranging from 8 to 9.6 (Supplementary Data 2), depending on location. Groundwater temperature generally correlates with overburden with the exception of high inflow zones where the largest faults occur around tunnel meter (TM) 1306, TM-2848 and TM-4166 (Figure 2). An additional zone of high inflow is observed in a landslide scarp section between TM 300 − 400, consistent with earlier hydraulic studies (Masset and Loew, 2010; Halter et al., 2024). Nitrate was detected in 22 of the 57 DELOS locations with elevated concentrations observed within the high inflow zones surrounding TM-300, TM-1306, TM-2848, TM-3750, TM-3884 and TM-4166 and a maximum concentration of 3.5 ppm at TM-216 (Figure 2). The average, per-fracture electrical conductivity (EC) in DELOS spans 43.2 μS cm−1 (TM-1306) to 931.1 μS cm−1 (TM-901; Figure 2). Groundwaters exhibiting an EC in excess of 500 μS cm−1 are also enriched with millimolar levels of sulfate. In general, the EC measurements of all groundwater-filled fractures from tunnel meters 4, 200 − 5, 218 of DELOS exhibit very little variation throughout the 16 month hydrochemical monitoring period (mean ± standard deviation: 91 ± 12 μS cm^−1^). On the other hand, the EC throughout the first 2,000 DELOS tunnel meters is highly variable between fractures but relatively consistent within a fracture throughout the sampling period (Figure 2). Inter-fracture water isotope measurements also exhibit greater variability in the first 2,000 tunnel meters relative to the last 1,000 tunnel meters (Figure 2). It is worth noting that the last 1,000 tunnel meters of DELOS is likely influenced by the drainage effect of the ~15 km long Furka Base Tunnel (Amberg, 1983) and, therefore, the similarities in the hydrochemistry of these sites may be anthropogenically driven.

**Figure 3:**
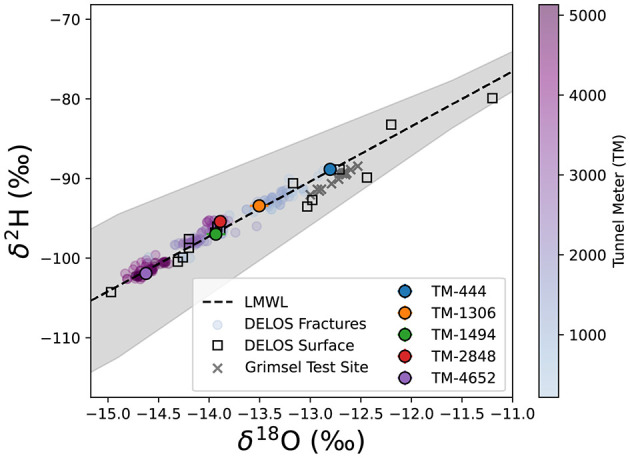
The water-derived δ^18^O (x-axis) and δ^2^H (y-axis) of surface waters and snow collected above DELOS (open squares) are shown with respect to an estimated local meteoric water line (LMWL, black dashed line surrounded by a 95% confidence interval illustrated as a gray box). DELOS fracture water samples (circles) are colored according to a blue-to-purple gradient that corresponds to the tunnel meter (TM) in which the sample was collected (see color bar). The 5 “long-term biological monitoring” locations are illustrated according to the site colors used throughout this manuscript. Information on the DELOS surface samples included in this plot are provided in Supplementary Figure 5, Supplementary Data 2.

All DELOS groundwater and DELOS surface waters follow the local meteoric water line (LMWL, Figure 3). With the exception of TM-1494 whose water isotope values are more similar to groundwater from DELOS fractures with over 400 m greater overburden, DELOS groundwater exhibits isotopic depletion as overburden increases (Figure 3). In general, the per-fracture DELOS groundwater isotope values were relatively stable throughout the monitoring period and did not reveal strong seasonal pattern (Figure 2). Previous water isotope measurements from the Grimsel Test Site (Schneeberger et al., 2017) also follow the DELOS LMWL and are most similar to the shallowest DELOS groundwater sites (Figure 3). It is worth noting that the nearby (< 15 km away), granite hosted Grimsel Test Site (GTS) is located approximately 1,730 m above sea level (masl) (Blechschmidt and Vomvoris, 2015) and the GTS water isotope samples are derived from sampling locations with an overburden of 420 − 520 m (Schneeberger et al., 2017). The surface elevation of DELOS groundwater samples with similar isotopic composition as the GTS samples is approximately 1,760 masl (overburden of 280 m).

### 3.2 DELOS microbial diversity and abundance

**Figure 4:**
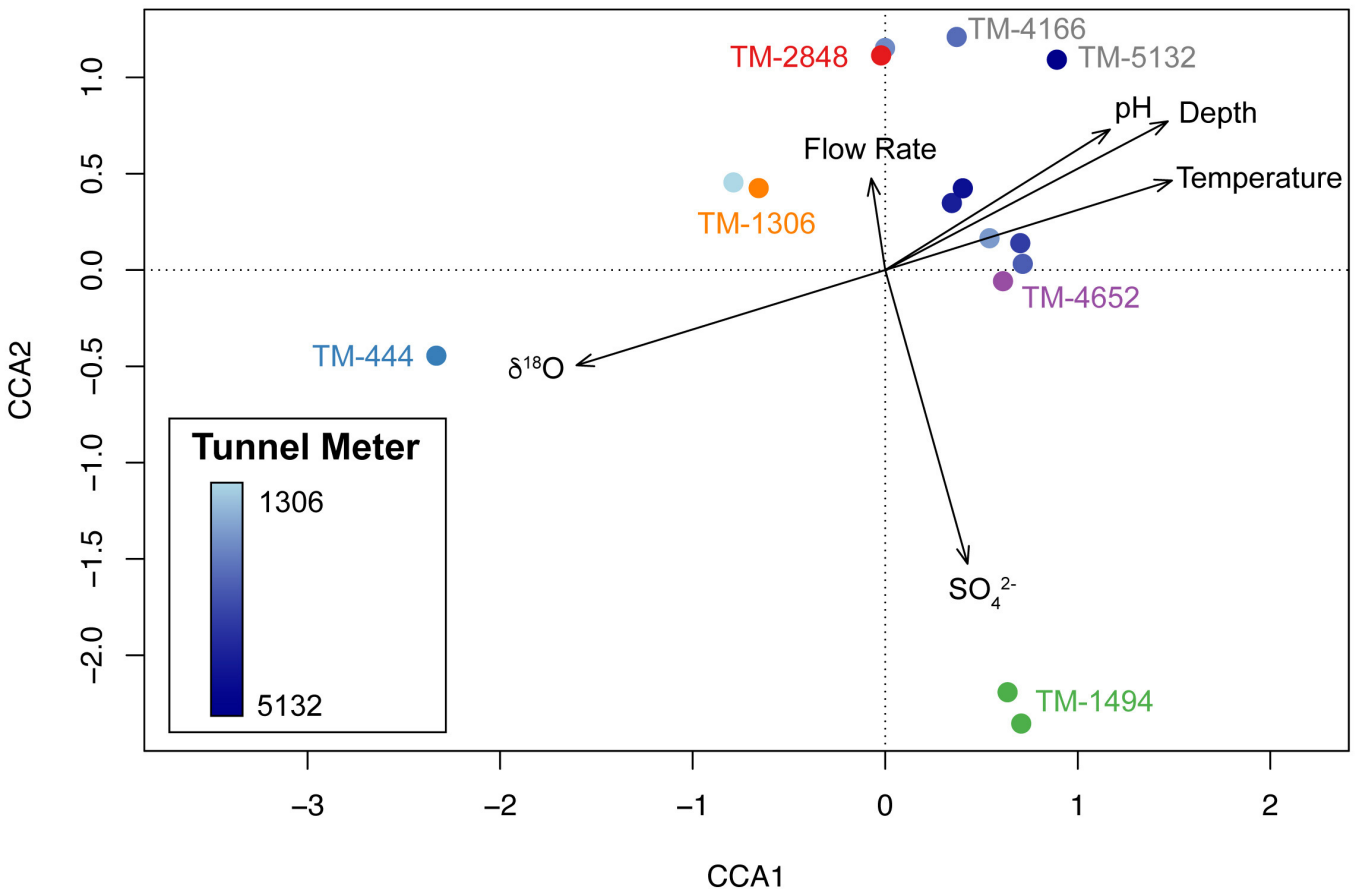
Ordination of the amplicon sequencing variant (ASV) taxonomic composition of DELOS fracture water samples collected in November and December 2020 (circles) along environmental gradients (black arrows) are displayed through a canonical correspondence analysis (CCA) plot. With the exception of long-term biological monitoring sites TM-444 (blue label), TM-1306 (orange), TM-1494 (green), TM-2848 (red) and TM-4652 (purple), samples are colored according to a light blue to dark blue gradient that corresponds to the tunnel meter (TM) in which the sample was collected.

**Figure 5:**
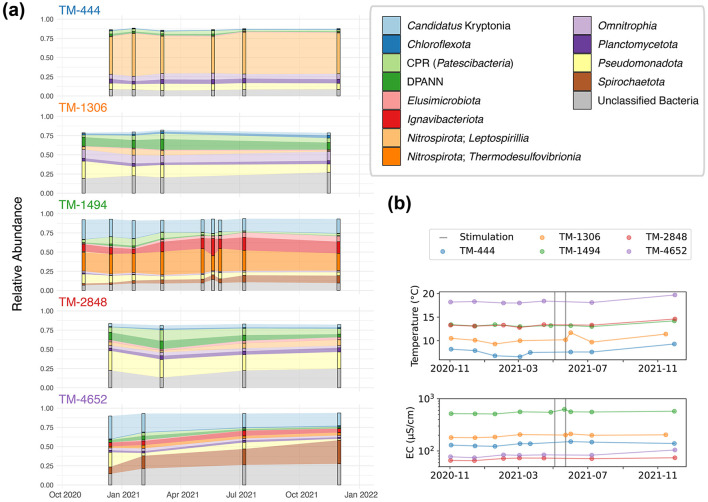
**(A)** The 16S rRNA gene amplicon based community composition of 5 DELOS fractures (TM-444, TM-1306, TM-1494, TM-2848, TM-4652) are shown. Each time point's microbial community is displayed as a bar with the observed change between sampling sites illustrated as the shading between bars. The taxonomic legend is displayed in the top right of the figure. Unfilled space between the top of each bar plot to 1.00 on the y-axis are taxonomic groups that are minor members of the community. **(B)** Changes in temperature (top) and electrical conductivity (EC, bottom) throughout the sampling period are shown for each sampling location. Vertical gray bars indicate the experimental period of 2 hydraulic stimulation experiments associated with the Bedretto Reservoir Project in May 2021.

14,508 unique amplicon sequencing variants (ASVs) spanning 64 phyla were identified throughout DELOS with the highest richness (2,944 ASVs; Shannon Diversity (*H*′): 5.8) observed in the November 2021 TM-1306 sample and the lowest richness (267 ASVs; *H*′ = 4.0) observed in the December 2020 sampling of TM-4166. DELOS microbial communities are dominated (85 to >99%) by bacteria. The most abundant bacterial phyla observed within DELOS are related to *Candidatus* Kryptonia (up to 30% at TM-4652), *Nitrospirota* (up to 55% at TM-444), *Pseudomonadota* (previously known as *Proteobacteria*; up to 70% at TM-2647) and *Spirochaetota* (up to 30% at TM-4652) (Supplementary Data 3). A large (>50%) population of chemolithotrophic *Leptospirillia* from the phylum *Nitrospirota* make the microbial community of the shallow, gneiss-hosted sample from TM-444 an outlier relative to other samples (Figure 4). Rather than *Leptospirillia*, other sites exhibiting large populations of *Nitrospirota* are represented by members of the nitrogen and sulfur cycling class *Thermodesulfovibrionia* (up to 30% at TM-1494, Figure 5). The DPANN archaeal superphylum dominates DELOS archaeal communities and members of this group are most abundant (up to 15% of the total microbial community) in samples from TM-1306, TM-2848 and TM-4447. Ultra-small bacteria within the Candidate Phylum Radiation (*Patescibacteria*) and bacterial class *Omnitrophia* within the *Verrucomicrobiota* phylum are also relatively abundant throughout DELOS, exhibiting their highest relative abundances at TM-4166 (up to 15%) and TM-4447 (up to 23%), respectively.

Fractures within the gneissic section of DELOS generally exhibit higher concentrations of 16S rRNA gene amplicon copies mL^−1^ (Q_25%_=7.6 × 10^3^, Q_75%_=3.3 × 10^4^) relative to granitic samples (Q_25%_=1.7 × 10^3^, Q_75%_=6.4 × 10^3^) (Supplementary Figure 3). An exception to this trend is observed at the granitic TM-2467 fracture (8.2 ± 2.1 × 10^4^ 16S rRNA gene amplicon copies mL^−1^). Here, a visible biofilm surrounds the fracture of TM-2647 (Supplementary Data 1) and may have contributed to the elevated copy numbers in the samples. Large populations of sulfur oxidizing *Pseudomonadota* related to *Sulfurifustis* (35%) and *Thiobacillus* (10%) that, in the case of *Thiobacillus*, have been linked to biofilm formation in the granite-hosted Äspö Hard Rock Laboratory (Lopez-Fernandez et al., 2023), further point to potential biofilm contamination within the TM-2647 sample. On the other hand, the fracture at TM-4652 intersects a quartz vein (Supplementary Data 1) and consistently exhibited the lowest concentrations (931 ± 378) of 16S rRNA gene amplicon copies mL^−1^ with a community largely composed of *Candidatus* Kryptonia and *Spirochaetota*.

Canonical correspondence analysis of 16S rRNA gene amplicon sequencing variants (ASVs) and environmental data from DELOS groundwater samples collected in November and December 2020 reveals that DELOS groundwater microbial communities are distributed along a δ^18^O, depth and temperature gradient that correlates to tunnel meter (Figure 4). As with the DELOS hydrochemical measurements, samples from the last 1,000 m exhibit more similar microbial communities than samples collected throughout the first 3,500 m of the tunnel (Figure 4). An exception to the otherwise tight clustering of samples from the last 1,000 m is a pH 9.0 sample with relatively high abundance (25%) of an uncultivated lineage (“Dadabacteria” also known as *Candidatus* Desulfobacterota) from TM-5132. Further separation of DELOS ecosystem types (ecotypes) are seen in the sulfate- and *Thermodesulfovibrionia*-enriched TM-1494 sample and high flow rate and DPANN/*Patescibacteria*-enriched samples (TM-1306, TM-2848, TM-4166). Together, these findings point to three major ecotypes in the DELOS groundwater system: (1) Shallow groundwater with low electrical conductivity enriched in *Leptospirillia*; (2) High-inflow fault zones enriched in ultra-small bacteria and archaea; (3) Bicarbonate-enriched waters predominantly represented throughout the last 1,000 tunnel meters and that are enriched in *Candidatus* Kryptonia and *Spirochaetota*.

Following the DELOS-wide survey in November and December of 2020, five fractures were selected for seasonal monitoring based on hydrochemical and sampling considerations. 16S rRNA gene analysis reveals that community membership is generally stable and that inter-group differences were significantly greater than intra-group differences (Figure 5, Supplementary Figure 4). TM-4652 exhibits the most phylum-level changes during the 12-month monitoring period, revealing a slow replacement of relatively abundant *Pseudomonadota* and *Patescibacteria* with *Spirochaetota* and *Ignavibacteriota*. A similar change in the abundance of these phyla is observed at TM-1494; however, the most dramatic change in taxonomic composition within this site occurs when the dominant population of *Thermodesulfovibrionia* decreases from 32% to 20% relative abundance from 5 − 21 May 2021. This decrease co-occurs with an increase in the relative abundance of *Ignavibacteriota* and two of unclassified bacterial ASVs that are also observed in TM-1494 throughout the various sampling time points and in TM-4652. This change in community composition follows the first hydraulic stimulation experiment in the Bedretto Reservoir Project's stimulation boreholes (ST, located near TM-2050 of BULGG) on 3–5 May 2021, seismic events near the TM-1494 fracture possibly related to the hydraulic stimulation on 6 May 2021, and seismic events near the TM-1494 fracture associated with heavy rainfall on 10 May 2021 (*personal communication*, BULGG). An increase in electrical conductivity that correlates to an increase in all major ion concentrations except magnesium is additionally observed at TM-1494 between 5 and 21 May 2021. A second hydraulic stimulation experiment that targeted a different depth range (interval) of the aforementioned experimental ST borehole was conducted from 22 − 24 May 2021. This experiment demonstrated a connection between the injection and production wells of the stimulation (Arnet, 2021) but did not result in seismic activity near the TM-1494 fault. The 1 June 2021 sampling of TM-1494 after the 22 − 24 May 2021 stimulation revealed that the fracture's hydrochemical and microbial community composition had recovered to its “baseline” state, indicating that the changes observed on 21 May were relatively short-lived (Figure 5). Interestingly, an increase in electrical conductivity along with an increase in temperature following the 22 − 24 May hydraulic stimulation can be seen in TM-1306 and may indicate that the two Bedretto Reservoir Project stimulation experiments affected two different regions of the Bedretto Reservoir (TM 2000-2100); however, a microbial sample was not collected from TM-1306 during this time period (Figure 5). Together, these observations indicate that both experimentally and naturally induced seismicity contribute to the temporal biodiversity and chemical patterns observed throughout DELOS.

### 3.3 DELOS groundwater and surface microbial communities exhibit minimal overlap

Three groundwater samples from the granite-hosted Grimsel Test Site (GTS, ~15 km from DELOS) and nine samples from DELOS surface waters (Figure 1) were collected for hydrochemical and microbial community analysis in spring and summer 2021, respectively. These samples represent water from two GTS experimental boreholes (“Pinkel” and “ISC Injection 2”), a GTS monitoring borehole (“SB80001”), the Ticino River, a high-altitude freshwater spring, four glacial lakes, and three samples (one above ice, two below ice) from the Witenwasseren glacier (Supplementary Data 2). Surface samples exhibited a lower mean temperature (6.8 ± 3.5°C) and pH (7.4 ± 0.6) than DELOS and GTS (Table 1). The electrical conductivity of the Ticino River is elevated (EC, 409 μS cm^−1^) relative to other surface sites, resulting in the high variance in EC across surface sites (Table 1). Groundwater collected from the GTS boreholes exhibit mean temperature, EC and 16S rRNA gene amplicon copies mL^−1^ measurements between the DELOS surface and DELOS groundwater samples (Table 1).

**Table 1:** The mean and standard deviation of temperature, pH, electrical conductivity and 16S rRNA gene amplicon copies per mL collected from DELOS groundwater, Grimsel Test Site groundwater and surface locations surrounding DELOS (DELOS surface).

	**DELOS groundwater**	**Grimsel Test Site**	**DELOS surface**
Temperature (°C)	14.1 ± 3.5	12.9 ± 0.4	6.8 ± 3.5
pH	9.0 ± 0.9	10.3 ± 0.4	7.4 ± 0.6
Electrical Conductivity (μS cm^−1^)	252 ± 250	108 ± 14	72 ± 144
16S rRNA copies (mL^−1^, Q_25%_)	2.0 × 10^3^	4.5 × 10^3^	1.0 × 10^6^
16S rRNA copies (mL^−1^, Q_75%_)	8.2 × 10^3^	5.0 × 10^5^	4.5 × 10^6^

Q_25%_ = 25% Quartile. Q_75%_ = 75% Quartile.

**Figure 6:**
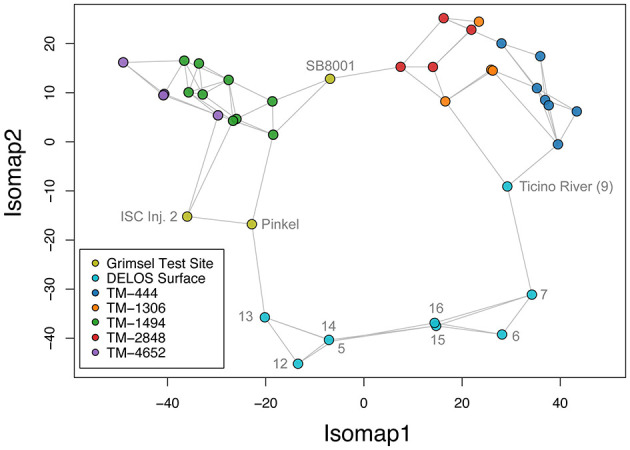
A three nearest neighbors isomap embedding of the rarefied phylum-level community composition of samples (points) from the DELOS time series (blue, orange, green, red, purple), surrounding surface waters (light blue), and nearby Grimsel Test Site (olive) is shown. Gray labels indicate sample names from the Grimsel Test Site and DELOS surface as described in the main text and Figure 1. Distances between samples were computed using the robust Aitchison metric and the gray lines illustrate the isomap-constructed neighborhood graph. Points that are connected and within close proximity to one another represent the microbial communities that are more similar.

**Figure 7:**
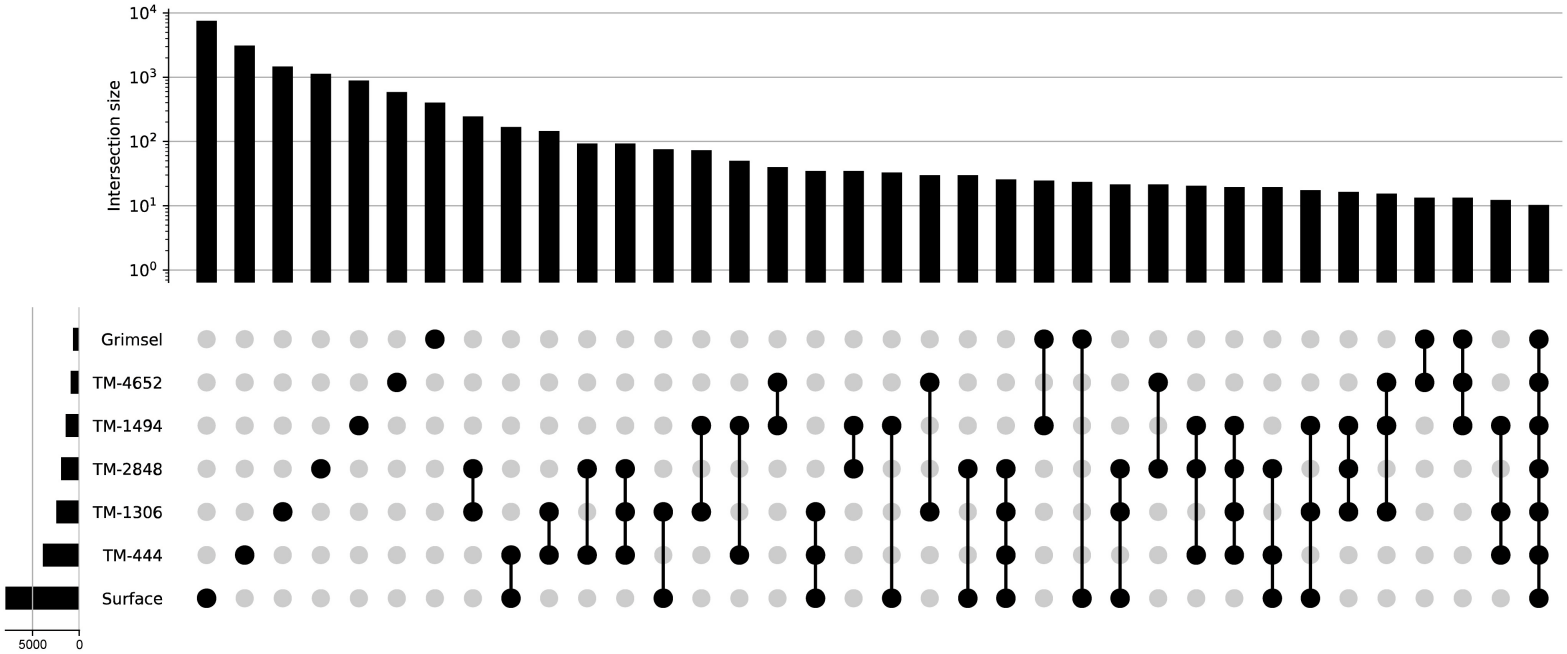
UpSet plot displaying the number of unique and shared amplicon sequencing variants (ASVs) between samples collected during the DELOS time series, from surface waters surrounding DELOS, and the Grimsel Test Site (x- and y-axis histograms). Black dots indicate the sampling locations for which the comparison of shared ASVs are being made. Comparisons for which there are less than 10 shared ASVs are not shown.

**Figure 8:**
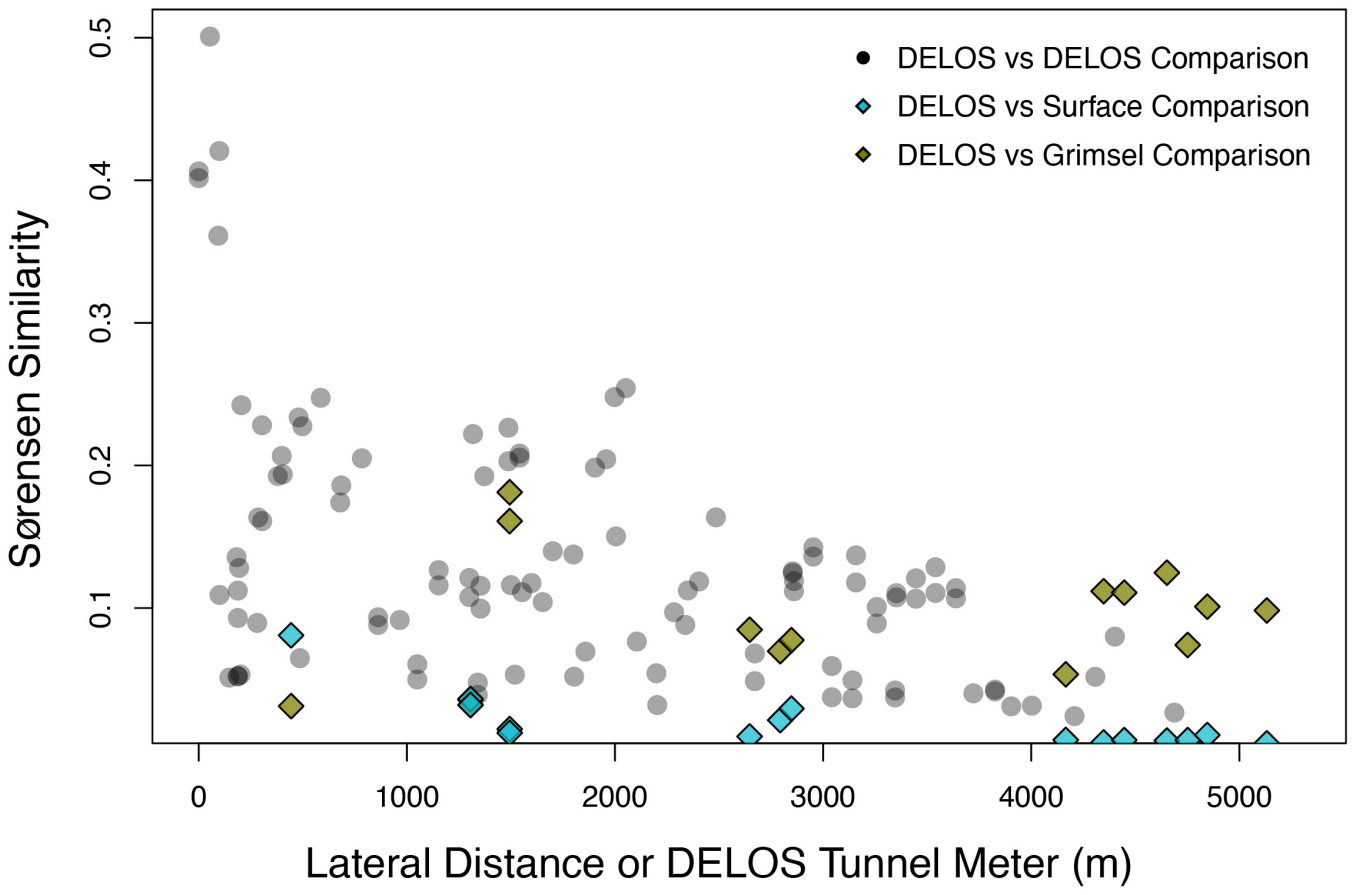
The amplicon sequencing variant (ASV)-level Sørensen Similarity (y-axis) between pairs of DELOS samples collected during November and December 2020 (black transparent circles) are shown with respect to the lateral tunnel distance between the pairs (x-axis). As a point of reference, pairwise comparisons between the individual DELOS samples with microbial communities from the monitoring borehole (SB80001) of Grimsel Test Site (olive diamonds) and the Ticino River (blue diamonds) are shown with respect to the tunnel meter in which the DELOS sample was collected (i.e., the far left olive and light blue diamonds represent comparisons between the December 2020 DELOS TM-444 sample and SB80001 and the December 2020 DELOS TM-444 sample and the Ticino River, respectively). SB80001 and the Ticino River were selected for comparison with DELOS since their microbial communities exhibited the greatest community overlap with DELOS samples.

The GTS groundwater samples captured three distinct microbial communities: (1) a *Desulfitobacteriia*-dominated (phylum *Bacillota*) community within the Grimsel *In-Situ* Stimulation and Circulation Experiment experimental borehole (Doetsch et al., 2018, “ISC Injection 2”) (2) a *Pseudomonadota*-dominated community composed primarily of *Hydrogenophaga* and *Rhodocyclaceae* in a radionuclide experimental borehole (Quinto et al., 2017, “Pinkel”) and (3) a *Pseudomonadota*- and *Thermodesulfovibrionia*-dominated community in the monitoring borehole (“SB80001”). Two Silva-defined bacterial phyla (*Caldisericota* and WS1) were identified in GTS at < 0.2% relative abundance but not DELOS and 29 phyla are found in DELOS but not GTS. On the other hand, 8 Silva-defined bacterial phyla (*Caldisericota, Campylobacterota, Fusobacteriota, Rhodothermota, Synergistota*, LCP-89, TX1A-33, and WS4) were identified in the surface samples surrounding DELOS but not DELOS, while 21 bacterial phyla were found in DELOS but not the surface water samples. At the phylum level, surface microbial communities were more similar to each other than DELOS groundwater communities (Figure 6) and there is very little ASV overlap between DELOS fractures, the DELOS surface, and nearby Grimsel Test Site (Figure 7). Furthermore, there is only a small tendency for nearby fractures to have more similar microbial communities (Sørensen Distance-Decay *R*^2^ = 0.2) with four of the five DELOS time series fractures exhibiting a greater similarity in community composition with GTS samples than the surrounding surface waters (Figure 8).

## 4 Discussion

**Figure 9:**
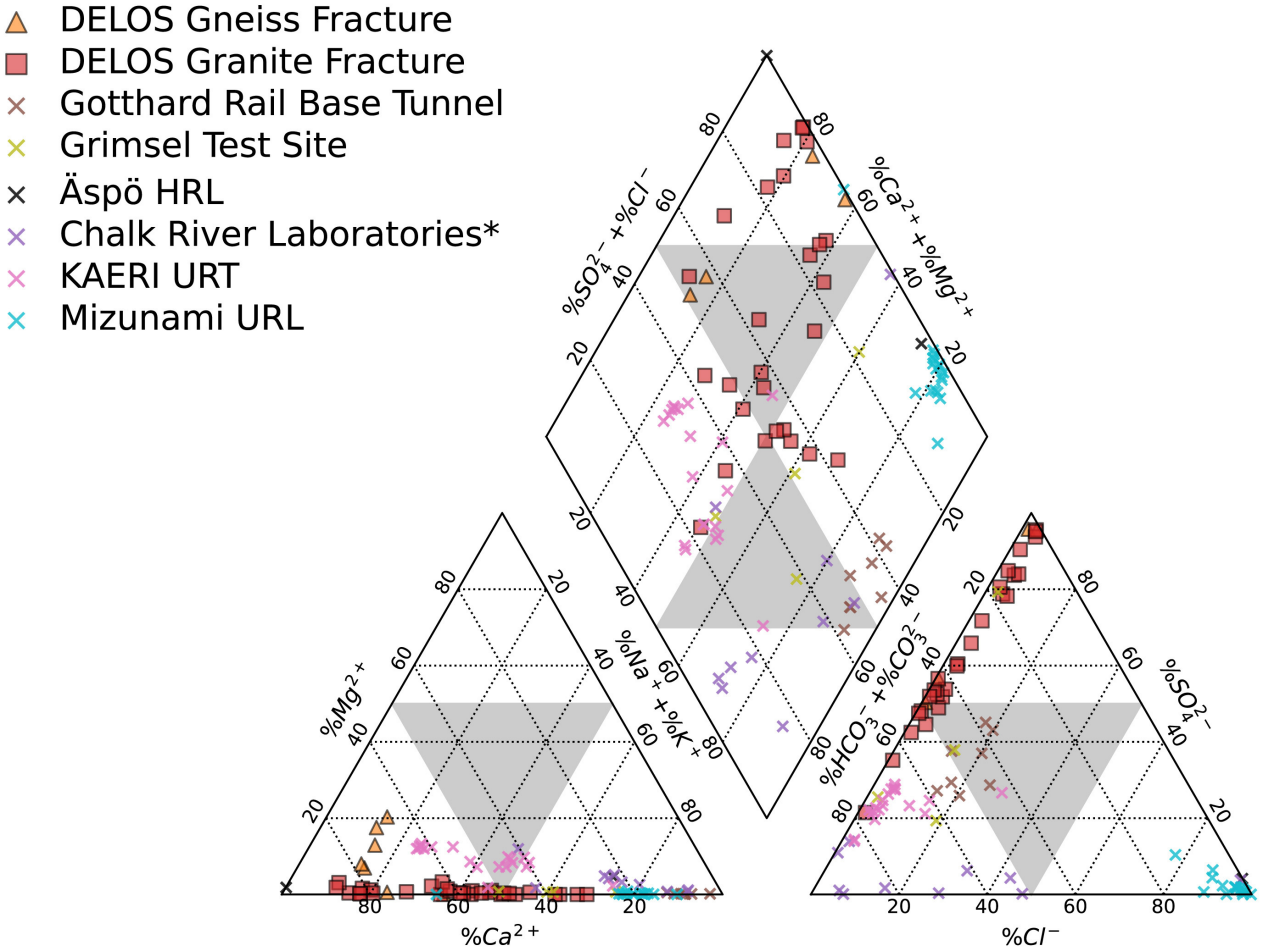
The mean annual composition of DELOS groundwaters derived from the gneissic (orange triangles) and granitic (red squares) sections of DELOS is displayed in a Piper diagram. Additional groundwater data from other subsurface laboratories and tunnels hosted in gneiss and/or granite (colored × 's) are shown. Data is compiled from the Gotthard Rail Base Tunnel (Switzerland) (Bucher and Stober, 2010), Grimsel Test Site (Swizerland) (Konno et al., 2013), Äspö Hard Rock Laboratory (HRL, Sweden) (Pedersen, 1997), Chalk Research Laboratories (Canada) (Beaton et al., 2016), Korea Atomic Energy Research Institute (KAERI) Underground Research Tunnel (URT) (Ryu et al., 2012), and Mizunami Underground Research Laboratory (URL; Japan) (Iwatsuki et al., 2005). The large gray triangles indicate the separation between water types. ⋆ = no K^+^ data provided for this study.

The BedrettoLab Deep Life Observatory (DELOS) provides a unique opportunity to study how hydrogeology affects subsurface microbiology. Unlike the majority of underground labs which make use of vertically excavated mines or deep boreholes (e.g. Hallbeck and Pedersen, 2008; Ino et al., 2016; Osburn et al., 2019), DELOS captures a 1, 600 meter depth gradient via a horizontally contiguous 5-km long tunnel through 2 main lithologies (gneiss-schist and granite) in the Alps. As with previous subsurface studies (e.g. Hallbeck and Pedersen, 2008; Ino et al., 2016; Osburn et al., 2019), the different geologies (gneiss-schist from TM-0 to TM-1138 and granite from TM-1138 to TM-5218) host hydrochemically and microbially distinct waters; however, within the same lithology, there are also high amounts of hydrochemical and biological variability correlated to depth, recharge, electrical conductivity and flow rate (Figures 2, 4, 6). Calcium- and sulfate-rich waters appear in the high EC sites (Figure 2) while the back half (>TM-2500) of the tunnel tends to have a larger sodium component. High inflow zones of the tunnel are depleted in sulfate and enriched in bicarbonate and nitrate (Figure 2, Supplementary Data 2). These bicarbonate-type waters are most similar to groundwater found in the granite-hosted Korea Atomic Energy Research Institute Underground Research Tunnel (KAERI URT; Ryu et al., 2012), Gotthard Rail Base Tunnel (< 15 km from DELOS; Bucher and Stober, 2010), and Grimsel Test Site (Konno et al., 2013). On the other hand, water from the Mizunami Underground Research Laboratory (Japan; Iwatsuki et al., 2005) and Chalk River Laboratory (Canada; Beaton et al., 2016) uniquely and respectively provide sodium chloride- and sodium bicarbonate-type end members among the granite underground laboratories reported in Figure 9.

Overall, DELOS exhibits a higher degree of hydrochemical diversity than reports of other granite-hosted underground labs (Figure 9) and, within DELOS, this hydrochemical heterogeneity is especially apparent within two granitic inflow zones (TM-1306 and TM-1494) separated by less than 200 m laterally and a difference in overburden of less than 50 m. Water from TM-1494 has a high sulfate concentration and a water isotope signature that is more similar to water encountered in deeper parts of DELOS. On the other hand, the water isotope signature and microbial community of TM-1306 is more similar to shallower parts of the tunnel and the Ticino River (Figure 3, Supplementary Figure 5). As stable water isotopes of oxygen and hydrogen are frequently used to trace groundwater and estimate recharge (Clark and Fritz, 2013; Blasch and Bryson, 2007), the hydrochemical and biological variation within the proximal but isotopically distinct fracture waters supports previous observations of groundwater origin and recharge shaping subsurface ecosystems (e.g. Ben Maamar et al., 2015; Hubalek et al., 2016; Yan et al., 2021). On the other hand, isotopically similar water from TM-1494 and TM-2848 (Figure 3) exhibit extremely different hydrochemical and microbial community compositions (Figure 5) while isotopically distinct water from TM-2848 and TM-4166 exhibit similar hydrochemistry and microbial community compositions (Figure 4, Supplementary Data 2, 3). The similarities between TM-2848 and TM-4166 appear to be correlated to the relatively high flow rates observed at these sites (Figures 2, 4) which also emerge near zones containing biotite-rich lamprophyres (Figure 2). Previous investigations into the microbial diversity of the metasedimentary Deep Mine Microbial Observatory (USA) have shown that mineralogy plays an important role in shaping subsurface hydrochemistry and microbial communities (Casar et al., 2020, 2021). A local enrichment of vanadium in the TM-2848 and TM-4166 groundwater and lamprophyres relative to other locations in DELOS (Supplementary Table 1) is suggestive of a similar connection between mineralogy, hydrochemistry and microbial diversity. As most groundwater vanadium studies surround sites with vanadium concentrations several orders of magnitude higher than DELOS (e.g. Xiao et al., 2017; Zhang et al., 2019, 2020a), additional work is needed to discern whether there is a significant relationship between vanadium and microbial diversity in DELOS.

Fracture flow rate, a proxy for hydraulic conductivity, may play an additional and understudied role in shaping subsurface ecosystems. DELOS fractures TM-1306, TM-2848 and TM-4166 occur within the 3 regions of highest inflow and all show an increased proportion of ultra-small bacteria and archaea relative to other sampling sites throughout DELOS. Previous studies have suggested that smaller cells are transported more easily than larger cells due to mechanical filtration (e.g. Gannon et al., 1991; Abu-Ashour et al., 1994); however, regions of DELOS with a lower flow rate and, thus a lower permeability and higher potential for filtering (Bense et al., 2013), do not exhibit an ultra-small microbial enrichment. Mechanical filtering is, therefore, not sufficient to explain the distribution of ultra-small microorganisms in this region and other factors such as groundwater turbulence may play a role. In shallow groundwater, an increase in the detachment of ultra-small bacteria from soils into groundwater during high infiltration periods has been observed (Herrmann et al., 2019, 2023). If similar processes control the abundance patterns of ultra-small microorganisms in DELOS, the increased turbulence associated within these highly permeable regions may similarly detach populations of ultra-small microorganisms from shallower biofilms and/or provide a medium to more easily carry small particles further.

Seasonal observation of DELOS fractures additionally indicates that groundwater mixing and/or alterations in flow paths can occur in DELOS (Figure 5). Research on the “critical zone” has found that changes in land cover and/or land use can change the biological and chemical properties of subsurface ecosystems and that extreme events such as heavy rainfall and earthquakes dominate the transfer of energy and matter from the surface to shallow (< 100 m below land surface) subsurface environments (Küsel et al., 2016; Skelton et al., 2019; Hosono et al., 2020). Seasonal mixing of more oxidizing shallow groundwater with deeper, more reducing fluids have also been observed and shown to introduce new substrates that stimulate the expansion of microorganisms into higher-energy niches (e.g. Magnabosco et al., 2018b; Bochet et al., 2020; Zhang et al., 2023) and human activities, such as tunnel construction and fluid injection, have been shown to alter groundwater flow paths and water chemistry (e.g. Banwart et al., 1996; Dong et al., 2014; Daly et al., 2016; Zhang et al., 2020b, 2022). Throughout the monitoring period, very minor seasonal changes were observed throughout DELOS (Figure 2); however, a measurable change in electrical conductivity and microbial community composition was observed in TM-1494 following a hydraulic stimulation experiment and rainfall-associated seismic events near the main fault sampled at this location. Hydrochemical measurements taken throughout the tunnel during this period (April–June 2021) suggest that these events may have altered the groundwater flow paths (Figure 2; Arnet, 2021). In particular, the increase in electrical conductivity observed at TM-1306 and TM-1494 during this period may be stem from an increase in the contribution of higher EC groundwater from the “badboy” fault (TM-1993, EC = 707 ± 64 μS cm^−1^) to these fractures. Although biological samples from TM-1993 are not available for comparison, a related experimental strategy, known as a “flow test” involving a 10 month injection of fluids into a deep borehole of the metasedimentary Sanford Underground Research Facility, showed that groundwater can be displaced during injection and result in the replacement of pre-existing communities with new microorganisms via advection (Zhang et al., 2022). Together, these observations suggest that the hydraulic stimulation and/or seismic events caused temporary re-organization of TM-1494's groundwater flow paths, recovering to “baseline” conditions within a matter of weeks.

Although further monitoring is needed to fully describe the connectivity of DELOS and the effect hydraulic stimulation experiments have on the hydrochemical and microbial community diversity patterns, correlated changes between monitored fractures provide new insights into the hydro-biogeological subsurface connectivity of a mountain block in a typical alpine watershed. Recently, a study of the 100,000 km^2^ Death Valley Regional Flow System (DVRFS, USA) identified shared taxa in hydrogeologically connected groundwater separated by over 100 km (Merino et al., 2022). At a more local scale, a small number of “core” species (97% OTUs) were reported to increase in relative abundance (from 6% up to 47%) as water flowed downstream of the preferential recharge area in the approximately 5 km Hainich Critical Zone Observatory (HCZO, Germany; Yan et al., 2020). Although the observations and relative proportions of core taxa in the DVRFS, HCZO and DELOS are not directly comparable due to differences in filtration volume and sequencing depth, only 2 ASVs were identified in all DELOS sampling locations from the November and December 2020 time points. These ASVs account for 0.9 ± 0.7% of each samples community with the highest relative abundance of these ASVs (2.5%) in TM-1494. Interestingly, despite exhibiting extremely different hydrochemical compositions, DELOS groundwater sampling location TM-1494 and GTS borehole SB80001 share the most ASVs. These ASVs are “rare”, low abundance taxa whose presence in both systems may indicate a similar source of water and microorganisms into these systems. Importantly, neither DELOS TM-1494 or GTS borehole SB80001 have been utilized for hydraulic stimulation experiments; however, these kinds of experiments have been performed in other boreholes within DELOS and GTS (Gischig et al., 2020; Ma et al., 2022) and, therefore, the sampling locations may experience similar changes in hydrochemical and microbiological compositions as a result of the injection procedures associated with hydraulic stimulation (e.g. Zhang et al., 2022). Ultimately, the subsurface fractures that exhibit the highest degrees of microbial community overlap tend to correlate with both hydrochemical and hydrogeologic features in DELOS, providing a new insights into the subsurface connectivity of the Riale di Ronco Alpine catchment.

## 5 Conclusions

Situated in the Alpine catchment of Riale di Ronco, Switzerland, the BedrettoLab Deep Life Observatory (DELOS) provides a new window into the continental subsurface biosphere by capturing an approximately 1, 500 m depth gradient over 5.2 km, largely within the same host rock. Despite the proximity and geologic similarity of subsurface sampling locations within DELOS, a wide range of hydrochemical and microbial community compositions are observed. Despite this variability, broad ecotypes can still be defined. These ecotypes are largely differentiated by the concentration of sulfate, bicarbonate and ultra-small bacteria and archaea. Additional biogeochemical monitoring of DELOS over a one year period further demonstrates the utility of coupling microbial diversity and hydrochemical studies to help inform hydraulic connectivity and groundwater flow. In particular, hydrochemical responses in TM-1306 and TM-1494 following seismicity suggest that groundwater from these locations experience mixing with warmer, sulfate-rich water but recover to their baseline hydrochemical and biological^1^ compositions within three weeks (Figure 2). Taken together, the spatial and temporal heterogeneity of DELOS provide a first look at the hydrochemical and biological diversity within DELOS and provide new insights in the groundwater history, local mineralogy, and hydraulic conductivity of a subsurface Alpine environment.

## Data Availability

The datasets presented in this study can be found in online repositories. The names of the repository/repositories and accession number(s) can be found below: https://www.ncbi.nlm.nih.gov/, PRJNA1181539. The data and code is available via https://github.com/GeobiologyLab/DELOS-2021-time-series.

## Footnotes

^1^Microbial community data for TM-1306 during the hydraulic stimulation was not collected.

## Supplementary material

Supplementary Data 1. Catalog of DELOS sampling sites.

Supplementary Data 2. Environmental and sequencing metadata.

Supplementary Data 3. 16S rRNA gene Amplicon Sequencing Variant and Annotation Table, available on https://github.com/GeobiologyLab/DELOS-2021-time-series.

Supplementary Table 1. A selection of DELOS granitic groundwater trace metal concentrations.

Supplementary Figure 1. The standard curve produced for the qPCR analysis of this study.

Supplementary Figure 2. The melt curve of the qPCR analysis of this study.

Supplementary Figure 3. DELOS 16S rRNA gene amplicon copies per mL.

Supplementary Figure 4. Analysis of Similarities (ANOSIM) of DELOS groundwater 1-year time series samples.

Supplementary Figure 5. Extended version of the water isotope plot of Figure 3.
