# Supplementary material for: Spatial and temporal groundwater biogeochemical variability help inform subsurface connectivity within a high-altitude Alpine catchment (Riale di Ronco, Switzerland)
Source: Front Microbiol. 2025 Feb 25;16:1522714. doi: 10.3389/fmicb.2025.1522714 (PMC11897985; doi:10.3389/fmicb.2025.1522714)
Supplement: Supplementary Table 1. — A selection of DELOS granitic groundwater trace metal concentrations. [file Data_Sheet_1.pdf]

Supplementary Materials:

Spatial and Temporal Groundwater Biogeochemical Variability Help Inform Subsurface Connectivity within a high-altitude Alpine catchment (Riale di Ronco, Switzerland)

Andrew S. Acciardo[1], Moira Arnet[1], Nima Gholizadeh Doonechaly[1,2], Alberto Ceccato[1], Paula Rodriguez[1], Hoang N.H. Tran[1], Quinn Wenning[1,3], Eric Zimmerman[1], Marian Hertrich[1], Bernard Brixel[1,4], Cara Magnabosco[1,*]

[1] ETH Department of Earth and Planetary Sciences, Zurich, Switzerland
[2] Centre for Hydrogeology and Geothermics (CHYN), Neuch ˆatel, Switzerland
[3] Current Address: Deloitte, Zurich, Switzerland
[4] Current Address: Cantonal Office of the Environment, Geneva, Switzerland

Correspondence*:
Cara Magnabosco
cara.magnabosco@eaps.ethz.ch

**Location**	**TM-4652**	**TM-4166**	**TM-2848**	**TM-1494**	**TM-1306**
**^{31}P (μM)**	9.06	20.58	22.88	14.73	24.77
**^{47}Ti (nM)**	1.77	0.56	0.77	4.37	1.54
**^{51}V (nM)**	0.28	13.26	22.05	0.36	1.60
**^{52}Cr (nM)**	0.43	0.50	0.71	0.55	1.56
**^{55}Mn (nM)**	4.15	2.28	2.05	293.65	7.61
**^{56}Fe (μM)**	32.44	2.62	6.68	30.18	20.95
**^{59}Co (nM)**	b.d.	0.01	0.02	0.09	0.08
**^{60}Ni (nM)**	0.67	0.21	0.33	1.67	1.01
**^{63}Cu (nM)**	0.73	0.32	0.25	0.50	0.63
**^{95}Mo (nM)**	411.69	226.19	450.04	619.31	357.89

Supplementary Table 1. A selection of DELOS granitic groundwater trace metal concentrations measured by inductively-coupled plasma mass spectrometry (ICP-MS, ThermoScientific Element XR) following the procedure outlined in Vance et al., 2016.

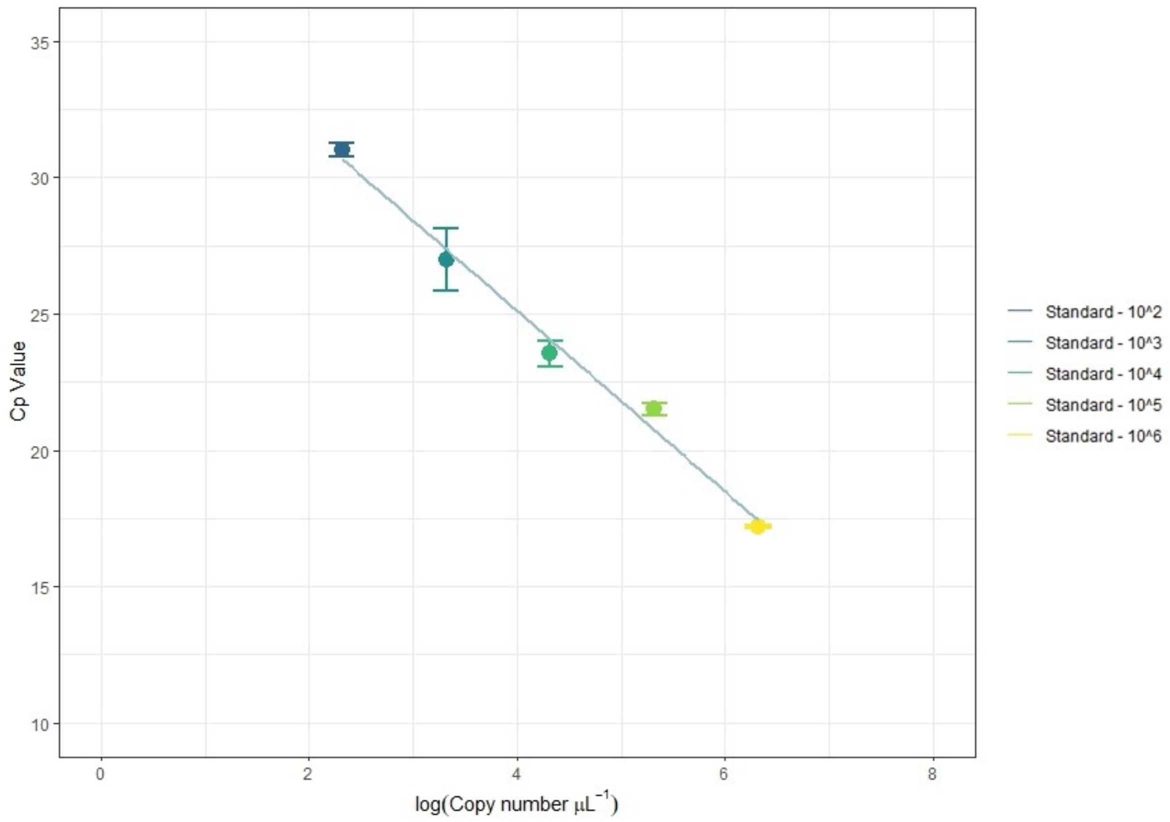

Supplementary Figure 1. Quantitative PCR (qPCR) standard curve. A consensus 16S rRNA gene sequence was determined and synthetic standards were prepared according to Han et al., 2023, whereby a region of the consensus sequence based on the 515F and 926R primer pair was selected and used to create the synthetic construct. A stock at a concentration of 2.088×10^7 copies μL^{-1} was first diluted to 2.088×10^6 copies μL^{-1} which would serve as the first standard. This was then serially diluted to a concentration of 2.088×10^2 copies μL^{-1}. Reactions for each dilution were prepared in triplicate and run on the LightCycler 480 (see Methods). The blue line indicates the standard curve ($y = -3.316x + 38.378$) with an R^2 value of 0.99.

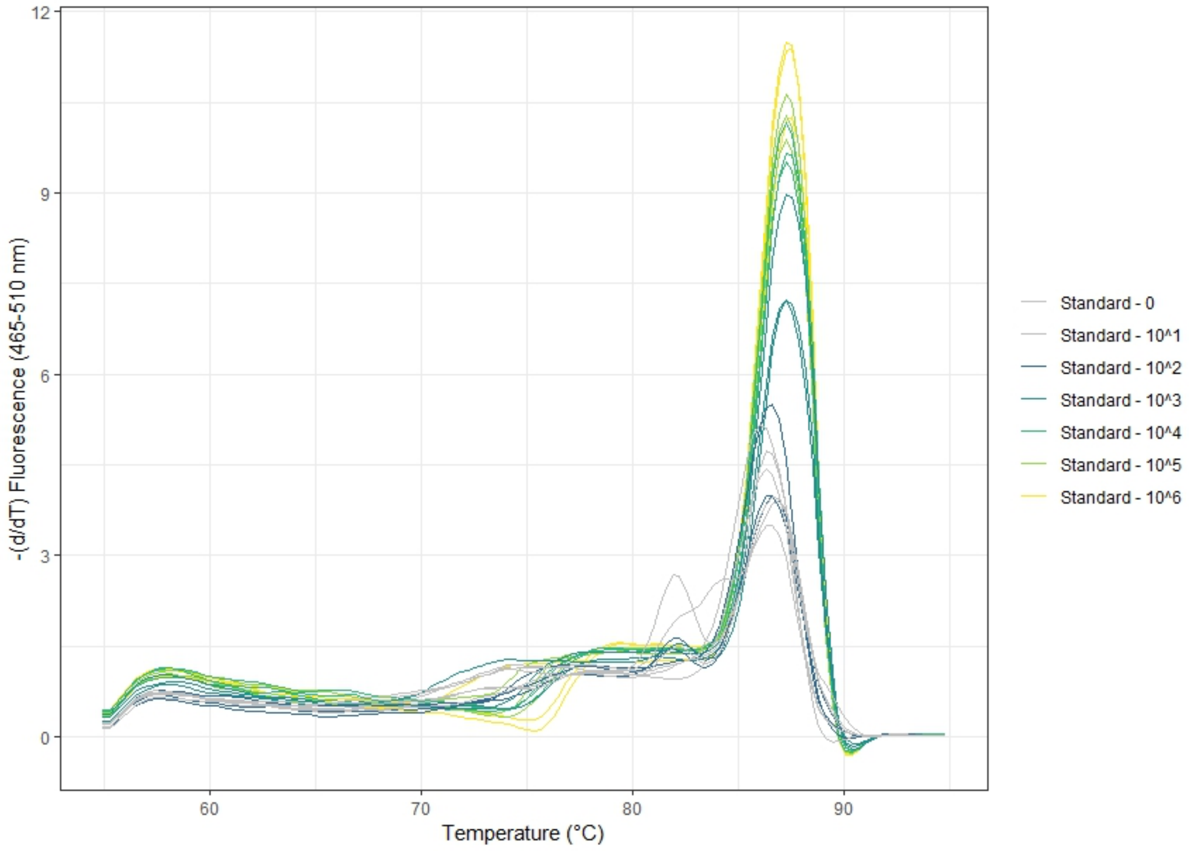

Supplementary Figure 2. Melt curve of qPCR standards. A melt curve plot displaying the negative first derivative of fluorescence with respect to temperature for qPCR standards, measured between 465-510 nm. Each line represents the melt curve for a standard, with triplicates of each standard shown in the same color. Peaks in the plot correspond to the temperatures at which the DNA dissociates, highlighting the specific melting points of different sequences. This data is used to verify the specificity of the PCR reactions. The bimodal and/or unsharp peaks in the melt curves of the low concentration standards, highlight the limit of detection for this method (10^2 copies μL^{-1}). The units for each standard are copies μL^{-1}.

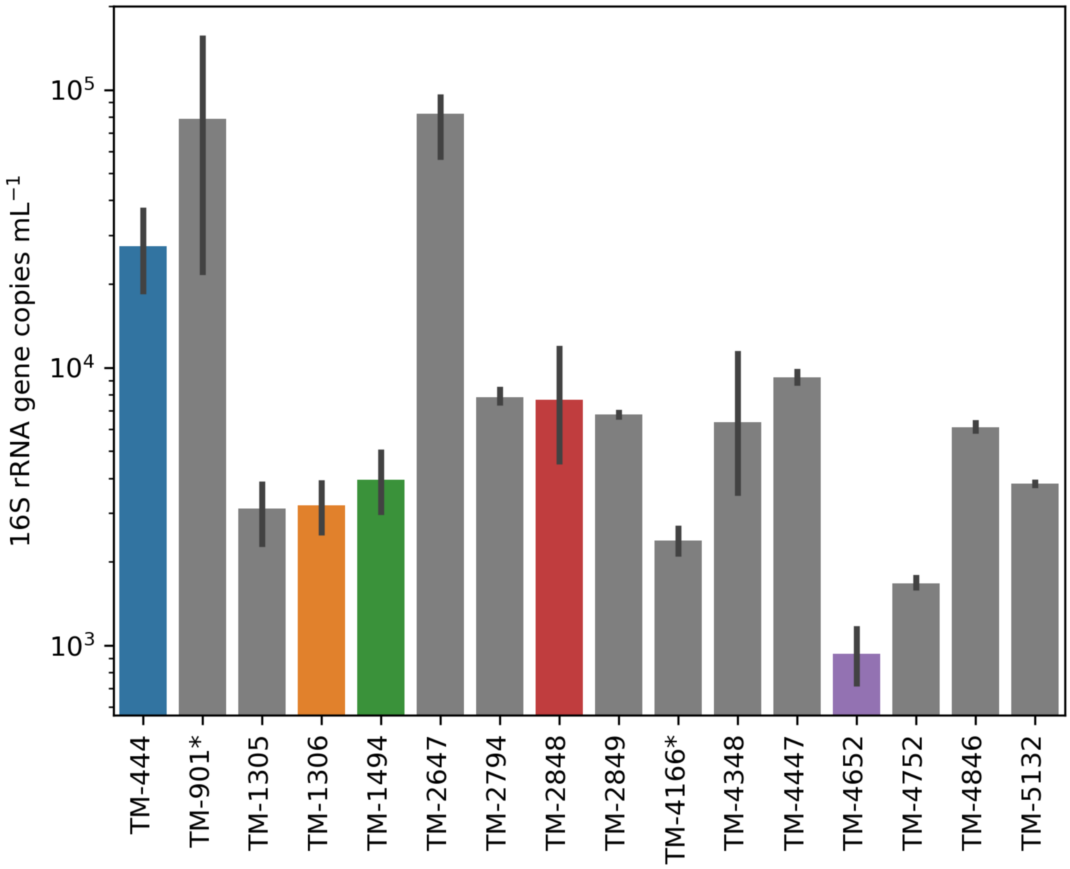

Supplementary Figure 3. 16S rRNA gene amplicon copies per mL (y-axis) as shown per sampling site (x-axis). Locations that were only sampled in 2020 are colored in gray with the standard deviation (black vertical lines) derived from triplicate quantitative PCR measurements of the sample. The * after TM-901 and TM-4166 indicates that 2 DNA samples were collected for these locations whereas other sampling locations colored in gray. Locations included in the 1 year time series are colored in blue (TM-444), orange (TM-1306), green (TM-1494), red (TM-2848) and purple (TM-4652).

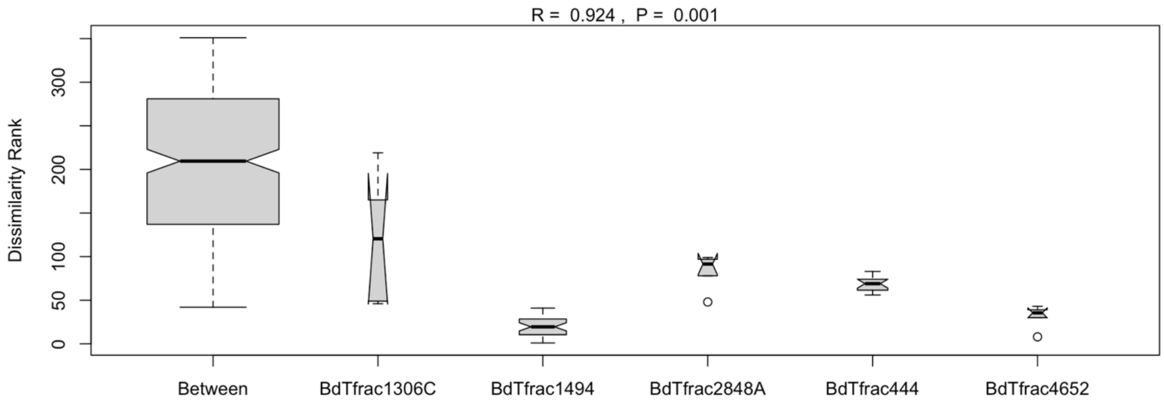

Supplementary Figure 4. Analysis of Similarities (ANOSIM) of DELOS groundwater 1-year time series samples. The dissimilarity rank is based on the Robust Aitchison SNV distance between samples. BdTfrac1306C = TM-1306, BdTfrac1494 = TM-1494, BdTfrac2848A = TM-2848, BdTfrac444 = TM-444, BdTfrac4652 = TM-4652.

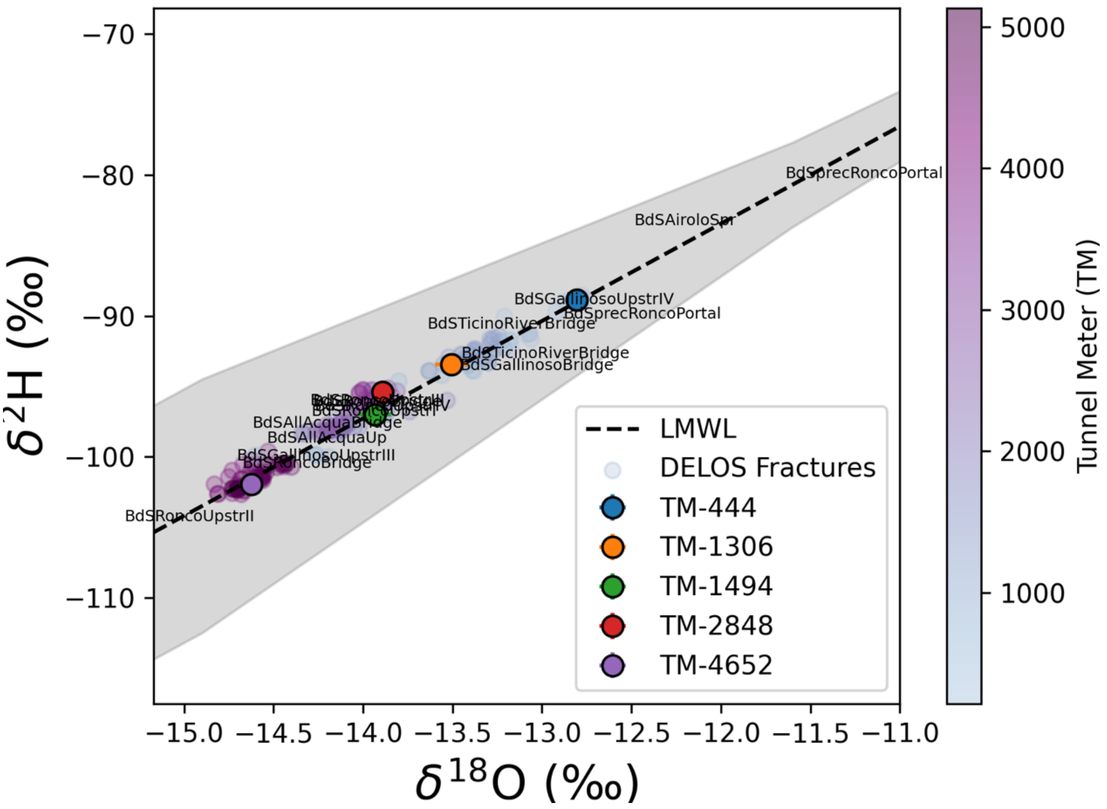

Supplementary Figure 5. Water isotope data obtained from DELOS Surface and Fracture Locations. Details on the surface samples listed in this figure can be found in Supplementary Data 2.

References

Vance, D., Matthews, A., Keech, A., Archer, C., Hudson, G., Pett-Ridge, J., & Chadwick, O. A. (2016). The behaviour of Cu and Zn isotopes during soil development: controls on the dissolved load of rivers. *Chemical Geology* 445, 36-53.
